# Early evidence (late 2^nd^ millennium BCE) of plant-based dyeing of textiles from Timna, Israel

**DOI:** 10.1371/journal.pone.0179014

**Published:** 2017-06-28

**Authors:** Naama Sukenik, David Iluz, Zohar Amar, Alexander Varvak, Vanessa Workman, Orit Shamir, Erez Ben-Yosef

**Affiliations:** 1 National Treasures Department, Israel Antiquities Authority, Jerusalem, Israel; 2 The Mina and Everard Goodman Faculty of Life Sciences, Bar Ilan University, Ramat-Gan, Israel; 3 The Martin (Szusz) Department of Land of Israel Studies and Archaeology, Bar Ilan University, Ramat-Gan, Israel; 4 Department of Archaeology and Ancient Near Eastern Cultures, Tel Aviv University, Tel-Aviv, Israel; Seoul National University College of Medicine, REPUBLIC OF KOREA

## Abstract

In this article, we focus on the analysis of dyed textile fragments uncovered at an early Iron Age (11^th^-10^th^ centuries BCE) copper smelting site during new excavations in the Timna Valley conducted by the Central Timna Valley (CTV) Project, as well as those found by the Arabah Expedition at the Hathor Temple (Site 200), dated to the Late Bronze/early Iron Ages (13^th^-11^th^ centuries BCE). Analysis by HPLC-DAD identified two organic dyestuffs, *Rubia tinctorum* L. and indigotin, from a plant source (probably *Isatis tinctoria* L.). They are among the earliest plants known in the dyeing craft and cultivated primarily for this purpose. This study provides the earliest evidence of textiles dyed utilizing a chemical dyeing process based on an industrial dyeing plant from the Levant. Moreover, our results shed new light on the society operating the copper mines at the time, suggesting the existence of an elite that was interested in these high quality textiles and invested efforts in procuring them by long-distance trade.

## Introduction

**Fig 1:**
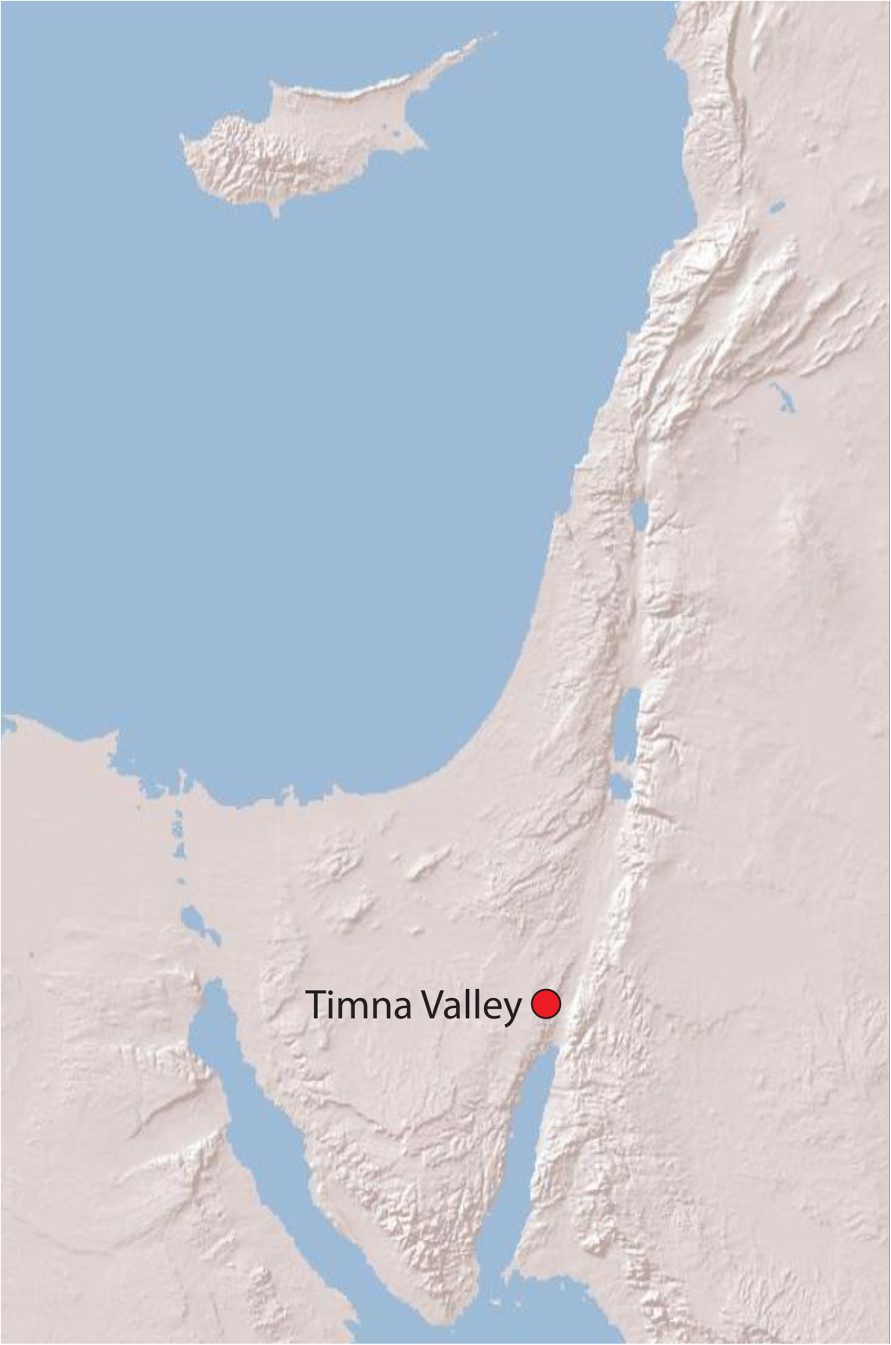
Map. The location of Timna Valley. Created using ArcGIS software by ESRI, based on a topographic model generated by NASA Earth Observatory (public domain).

In the course of the new excavations at Timna carried out as part of the Central Timna Valley (CTV) Project (Fig 1; [1]), many fragments of textiles and cordages were found: 116 fragments were uncovered during the 2013 and 2014 excavation seasons [2], and a few dozens of other textile fragments were uncovered in the successive 2015 and 2016 seasons. The largest assemblage was discovered at Site 34 and dated by radiocarbon to the early Iron Age (late 11^th^-10^th^ centuries BCE; [3]). The site, one of the largest copper smelting camps in the Timna Valley, is also commonly called “Slaves’ Hill”, following the description of Nelson Glueck [4], who considered its wall and location on a mesa surrounded by cliffs as a means to enclose forced laborers or slaves.

**Fig 2:**
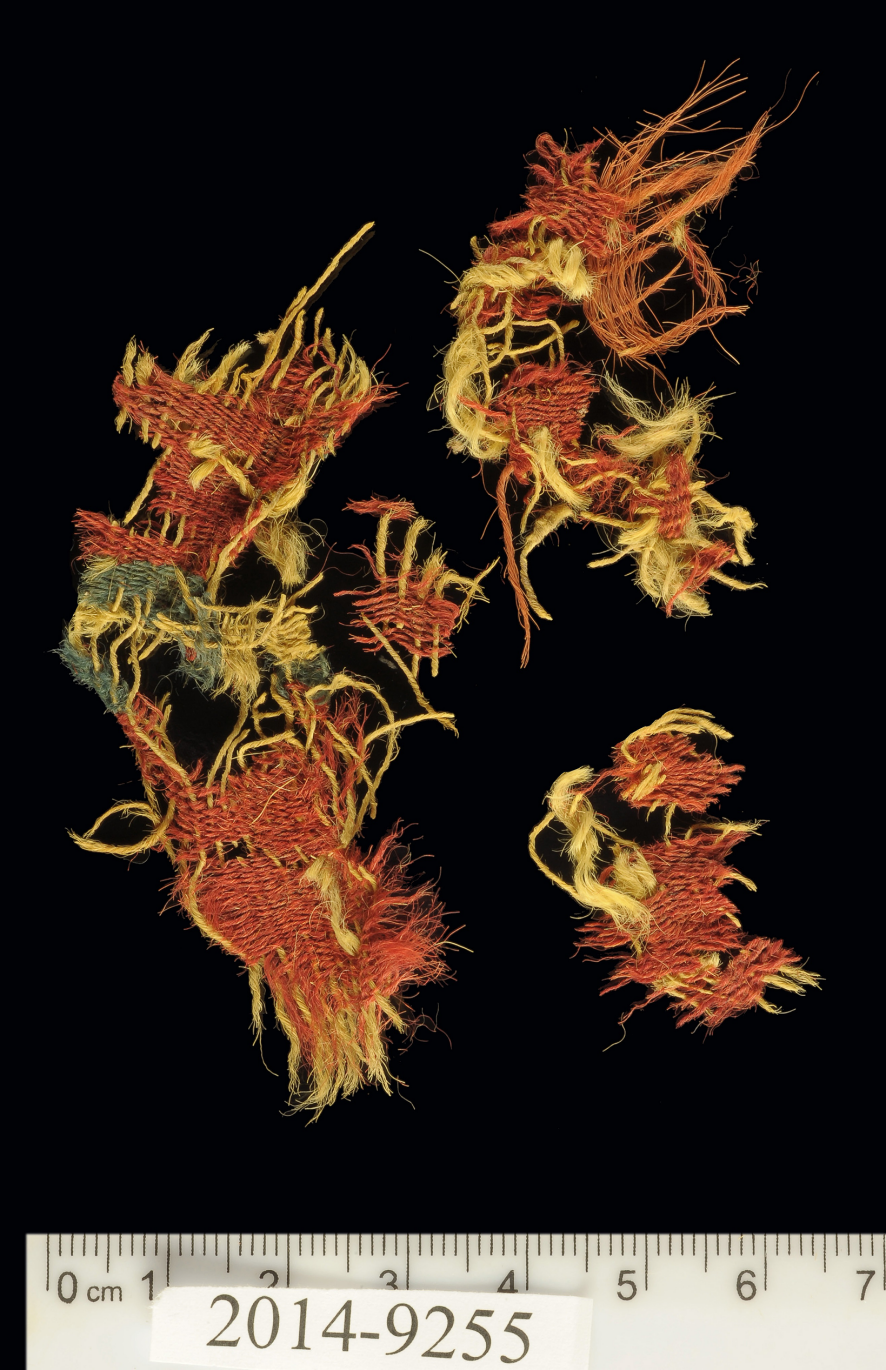
Wool textile (IAA no. 2014–9255). Textile decorated with red and blue bands. Photo by Clara Amit, courtesy of the Israel Antiquities Authority.

Archaeological textiles tend to be rare finds. Like any perishable organic material, they are subject to rapid decomposition in archaeological contexts and their preservation requires special conditions to prevent their destruction by microorganisms [5]. Generally, extremely dry or, alternatively, oxygen-deficient permanently wet environments such as in a peat bog, are the most conducive to the preservation of textiles in their original organic state [6]. This rare assemblage from Timna that was preserved as a result of the extremely dry climate in the region, provides a window into aspects of past societies that are inconspicuous in common archaeological research. The textiles discovered at Site 34 during the 2013 and 2014 seasons are made up of 83% wool fibers, while the remaining 17% is split between goat hair and bast fibers [2]. Moreover, 19 colored textiles deliberately dyed with red and blue hues were identified during analysis. It should be noted that although the preserved fragments are small, one can still see their original color even after 3000 years (Fig 2). The dyed textiles constitute a corpus of paramount importance, as there are very few parallels known to us today from the southern Levant, and their analysis provides a significant contribution to our knowledge of Iron Age garment and textile production technologies.

Textile-dyeing has been practiced since prehistoric times, using dyes extracted from both plant and animal sources, as well as inorganic materials. Modern analytical techniques make it possible to identify individual dye components from ancient textiles [7]. However, a thorough study of ancient dyeing industries requires an interdisciplinary approach, including, inter alia, archaeology, history, ethnography, geography, biology, and chemistry [8]. Such approach was applied in the current study in order to identify biological sources used for dyeing the Timna textiles and to assess their implications on our understanding of the society operating the copper mines in the turn of the 2^nd^ millennium BCE.

The aim of the current study was to identify the natural dyes and associated dyeing technologies used in the colored Timna textiles, as a basis for shedding new light on the ancient dyeing industry and the society operating the copper mines at the turn of the 1^st^ millennium BCE.

**Fig 3:**
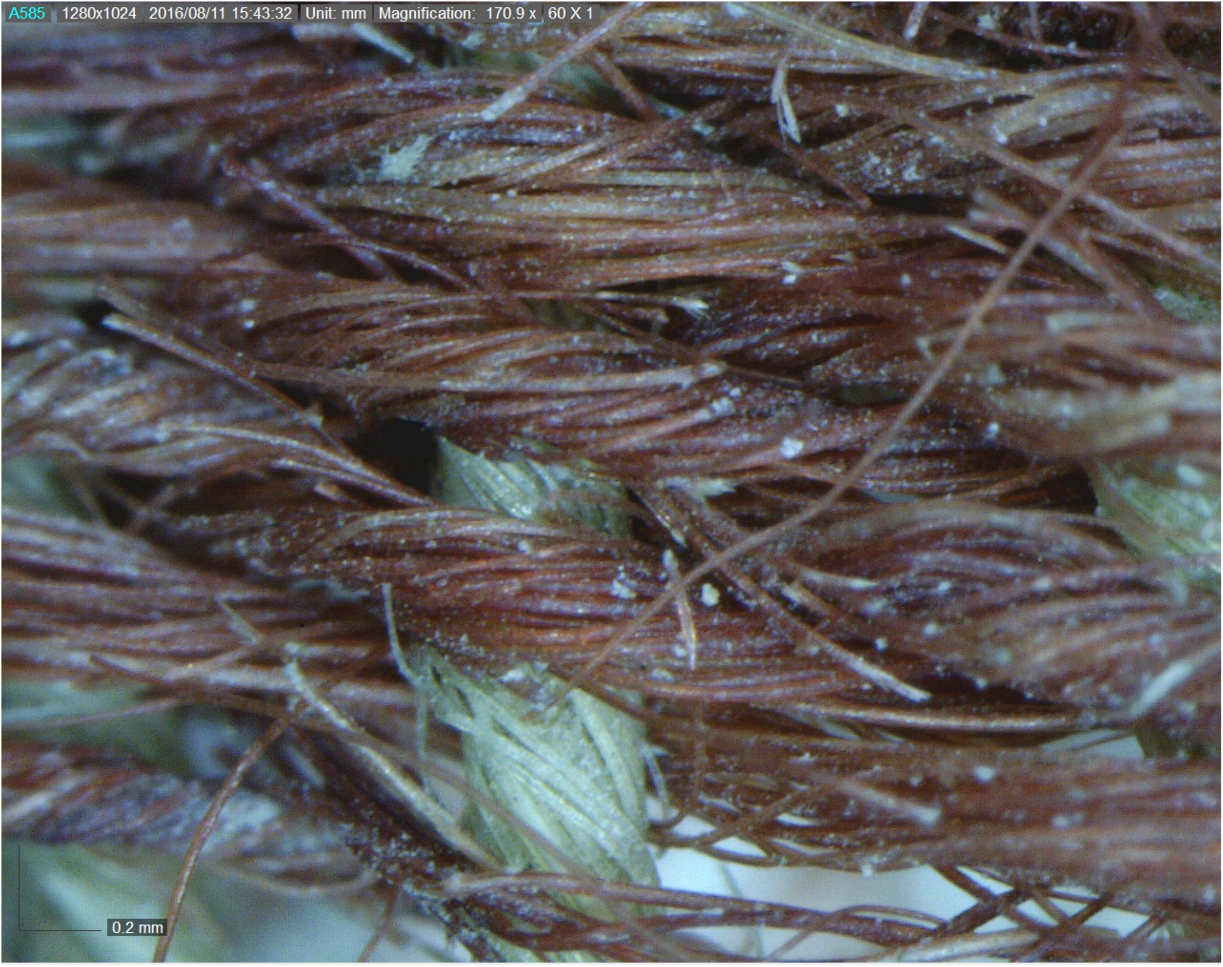
Part of textile (IAA no. 2014–9255). Textile with red threads. Photo by Naama Sukenik using Dino-Lite x170 magnification.

**Fig 4:**
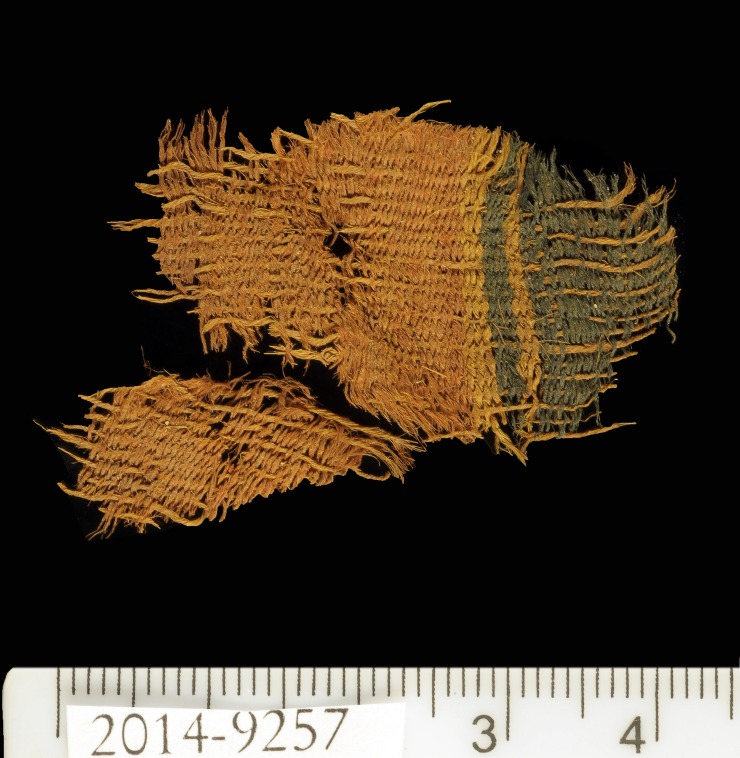
Wool textile (IAA no. 2014–9057) from Site 34. The textile was decorated with red and blue bands. Photo by Clara Amit, courtesy of the Israel Antiquities Authority.

**Fig 5:**
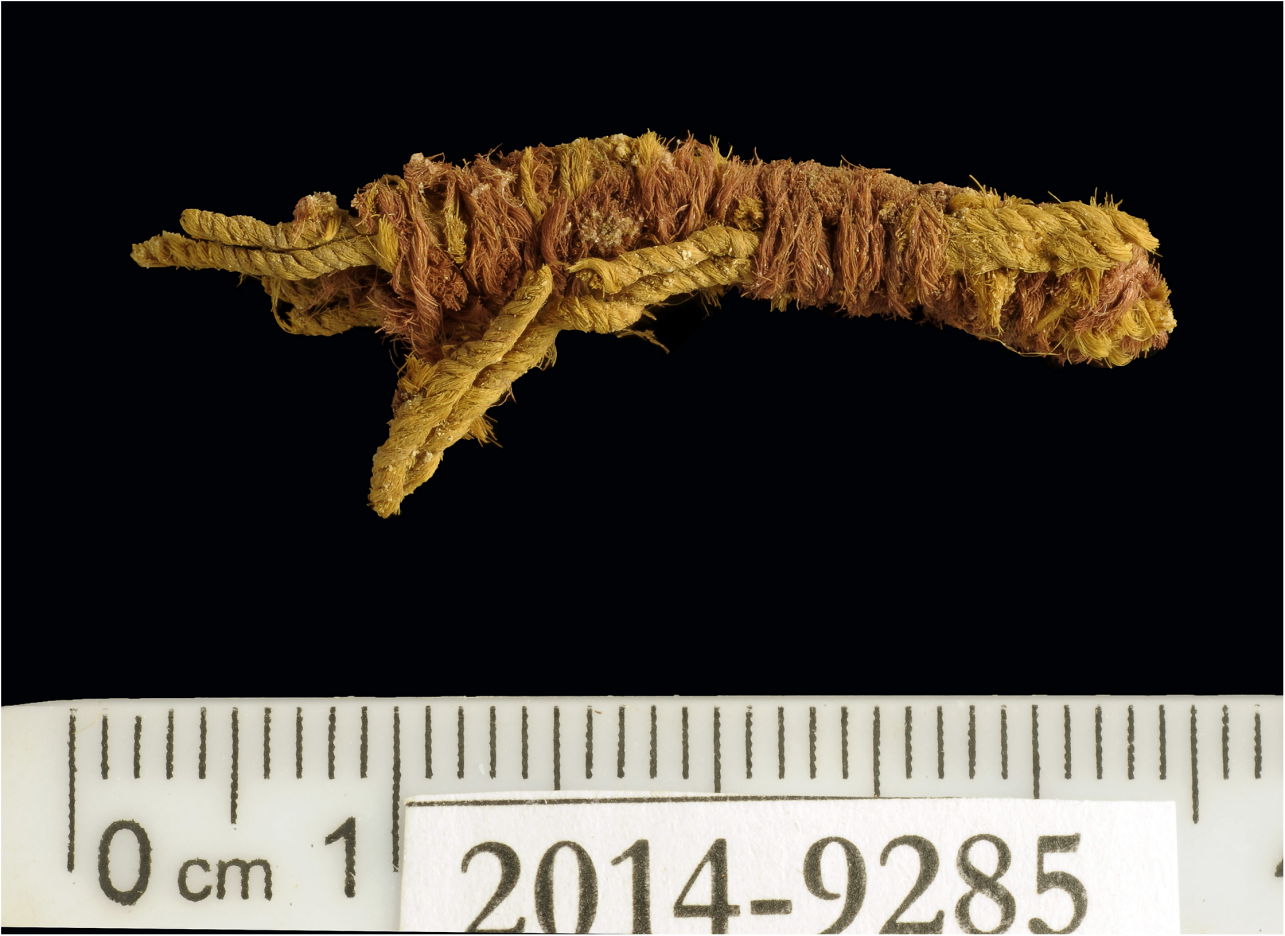
Tassel with red threads (IAA no. 2014–9285). Photo by Clara Amit, courtesy of the Israel Antiquities Authority.

The colored textiles are all made of wool, which was the best raw material for dyeing in the Levant prior to the introduction of cotton and silk in the latter period [9–12], due to the protein composition of the fiber that allows better absorption of the dye than that of the linen fiber [7,13]. A microscopic examination (using a Dino Lite x60—x170 magnifications), indicated that the fibers were dyed before they were spun into threads, a standard procedure in dyeing wool (Fig 3 [10,14]). The most common pattern in Timna was colored bands ([2] Figs 2 and 4), made by alternating or changing the color of the weft (horizontal) threads. Six decorative elements of tassels were uncovered at Site 34, one of them (IAA no. 2014–9285) was embellished with a red thread wrapped around tightly ([2] Fig 5). Although there are only few physical parallels of decorative tassels in the archeological records of the Bronze and Iron Age Levant (cf. an 8^th^ century BCE wrapped tassel from Kuntillat ‘Ajrud: [15]), it is widely attested for in artistic depictions (reliefs, paintings, and sculptures) throughout the Near East, and it seems that dyed tassels were a common ornamental element of clothing in ancient items. Reliefs on burial tombs of New-Kingdom Egyptian pharaohs, such as those of Seti I in the Amon Temple at Karnak [16] or of Ramses III at Medinat Habu, show depictions of persons commonly identified with various Semitic peoples (and, in particular, the Shasu nomads), who appear dressed in garments decorated with red and blue bands, with colored fringes on the garment edges [16,17].

Natural dyes can be classified as direct dyes, vat dyes, or mordant dyes, according to the method by which they are applied to textiles [8]. Direct dyes are water-soluble and bind directly to the fibers, while the vat dyes are insoluble organic pigments, such as indigoid dyes, that require a special dyeing method involving biochemical and photochemical reactions, as well as complex reduction and oxidation processes that necessitated the use of various additives and would have taken a number of days in ancient times [14,18]. Most of the dyestuff classified as mordant dyes require pretreatment to be fixed on the fibers by means of mordants, such as tannins and metal salts of aluminum, iron, and copper, which create a chemical bond between the dye and the fiber [14]. One of the most notable mordant used was alum (KAl (SO_4_)_2_^.^12H_2_O), which played a major role in the textile dyeing industry until the 19^th^ century CE [14].

The study of the dyes touches upon a variety of fields, including the quality of the dyeing industry, technological achievements, daily life, fashion, trade connections [5], and more. The newly discovered Timna dyed textiles join the previous findings from the Hathor Temple (Site 200) by Beno Rothenberg, where textiles dyed red, blue, and yellow were found [19]. The latter might be related to the worship of Hathor, as textiles were a favorable offering to this Egyptian goddess [20]. However, although Rothenberg associated the entire assemblage from the temple with the Egyptian presence during the Late Bronze Age [21], it is likely that some of the textiles originated from early Iron Age contexts, as suggested by the complex stratigraphy of the site [22]. At this time the site was still a temple, but dedicated to local deities [21,22].

## Materials and methods

In the current study we identified dyes by applying the HPLC- method, which has been widely used since 1985 for the identification of dyes found in archaeological artifacts [23]. Although the analysis itself is destructive, it can identify components that are present in minute amounts, thus only very little quantities of the tested substance are required, ensuring minimal damage. This method is, therefore, considered the most appropriate one for archaeological textiles and, at the same time, is reliable, with a high degree of accuracy and separation capability, qualities that are crucial for the identification of minor compounds in archeological textiles [7,24–27].

The identification of dyes was based on a database containing the “fingerprint” of the standards: known chemical compounds and dyed wool with known dyestuffs that were dyed and analyzed by the authors prior to the current Timna research. The standards were analyzed under the same conditions as the archaeological samples. In each test, a characteristic chromatogram was obtained, and the color compounds were identified by the particulars of their retention time (R_t_) and their characteristic absorbance spectra, including the wavelengths of the absorbance peak in the UV-visible spectrum (λmax). The dye substances were detected by comparing to the known chemical standards (S1 Appendix).

### Samples

**Fig 6:**
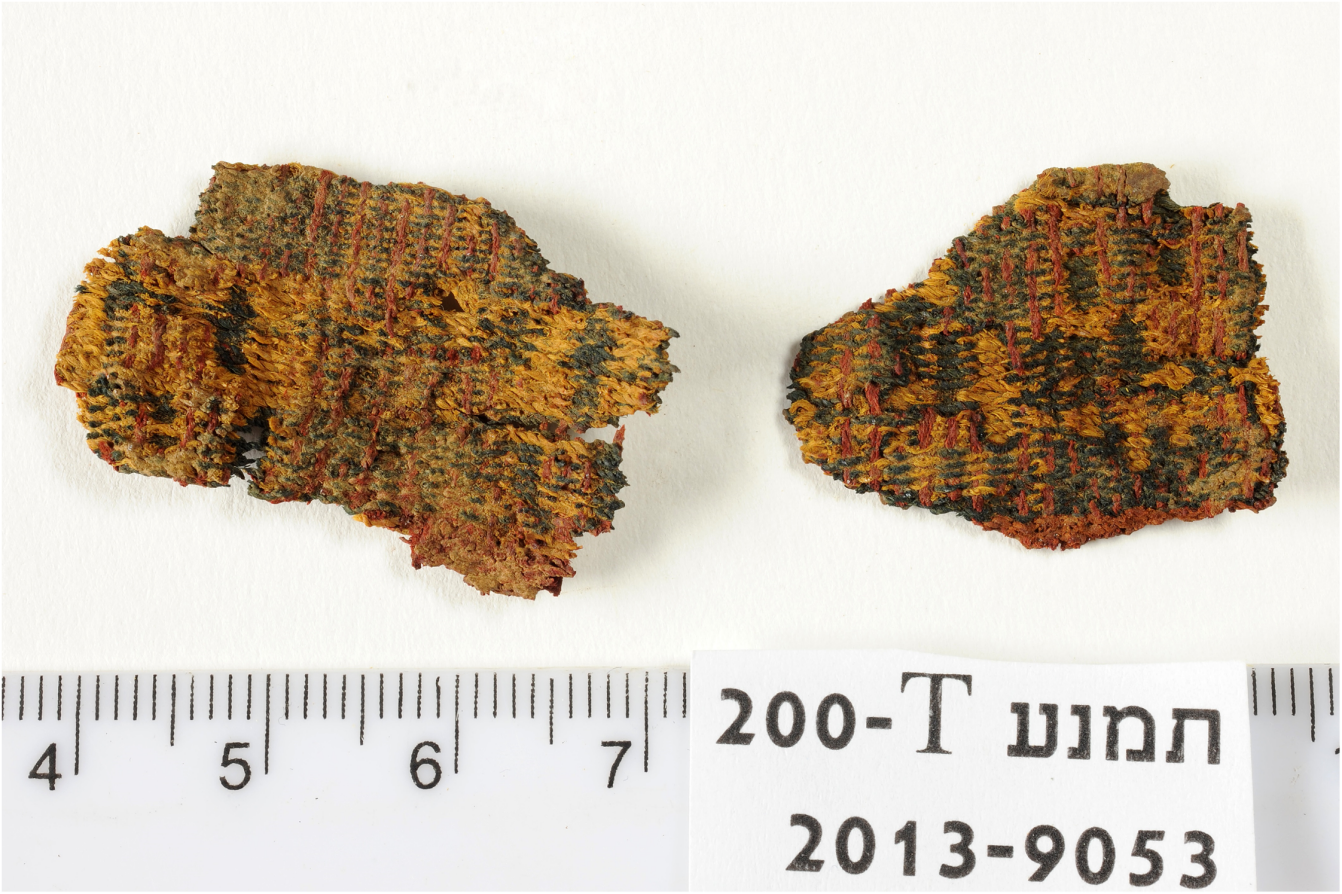
Fragments of wool textile (IAA no. 2013–9053). The textile woven in compound weave includes red, blue, and yellowish threads. Photo by Clara Amit, courtesy of the Israel Antiquities Authority.

**Fig 7:**
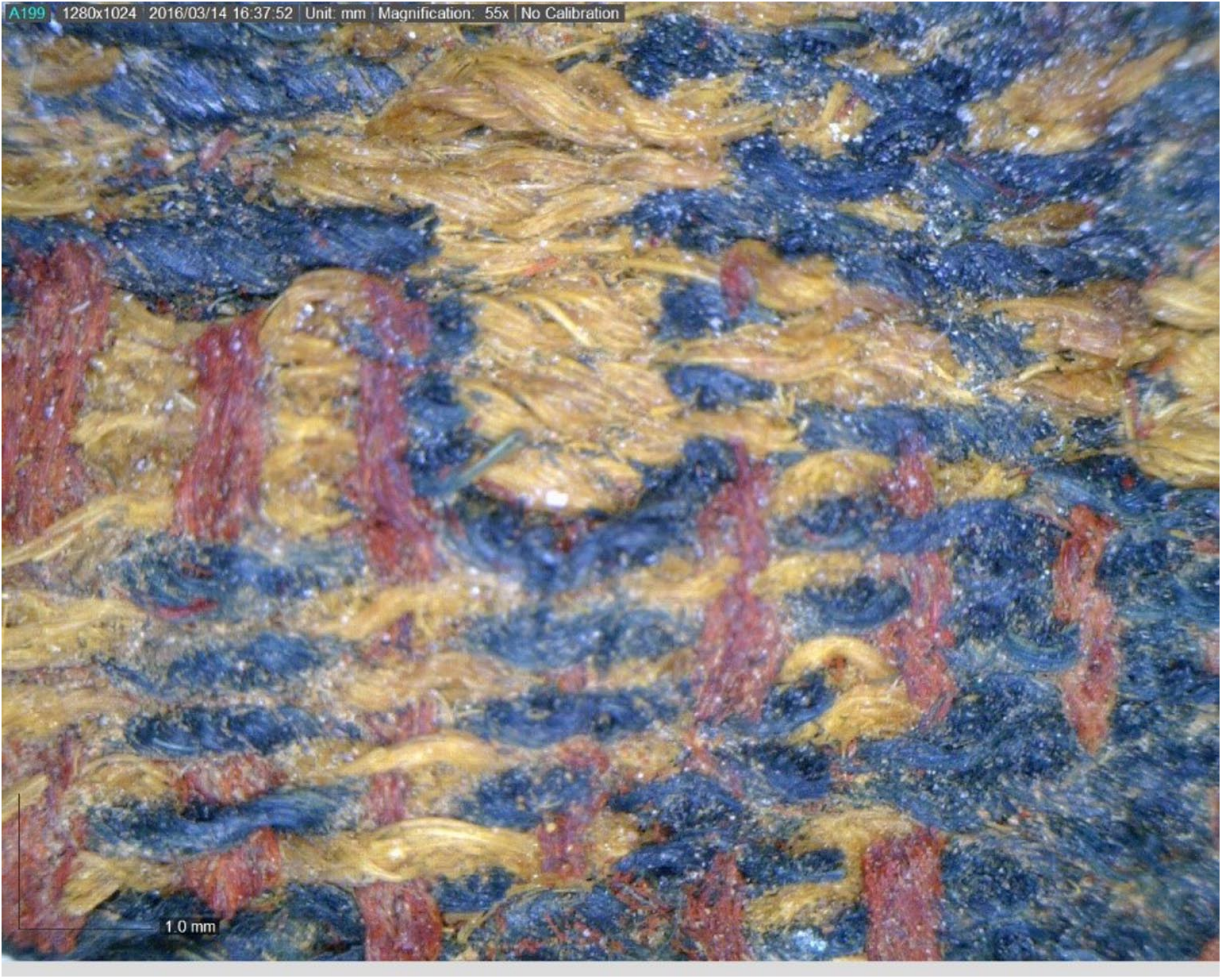
Part of textile (IAA no. 2013–9053). Compound textile includes red, blue, and yellowish threads. Photo by Naama Sukenik using Dino-Lite x70 magnification.

Seven 3-mg samples of red or blue fibers from five distinct textile fragments were analyzed as part of the current study (Table 1). All the samples are store at the Israel Antiquities Authority Facilities and available for researchers for further inspection. Samples 1–5 were taken from textiles found in well-defined contexts at Site 34 (samples 1–5), Another two samples (samples. 6–7) were taken from a textile fragment found in excavations carried out by Beno Rothenberg and the Arabah Expedition at the Hathor Temple (Site 200) (IAA textile no. 2013–9053, Figs 6 and 7, [21,28]). As part of the current project, this fragment was dated by radiocarbon to the late 13^th^ to mid-11^th^ centuries BCE (calibrated 2σ range, Table 2). For unknown reasons, these textiles were not published by Sheffer and Tidhar [19], who worked on the textiles from the Temple, and the exact contextual data have been lost. Preliminary investigation revealed that the textile was made by unusual (high-quality) compound weaving, and included red, blue, and yellowish threads (Figs 6 and 7).

**Table 1:** List of samples analyzed in the current study.

Number	IAA number	Color	Site/Area	Context (Locus / Basket)
**1**	2014-9255(R)	Red	34/19C	L.243/ B.502
**2**	2014-9255(G)	Green-blue	34/19C	L.243/ B.502
**3**	823476	Blue	34/GS	L.460/ B.5311
**4**	2014–9465	Blue	34/GS	L.456/ B.5331
**5**	2014–9240	Red	34/19A	L. L112/ B.112/1
**6.**	2013-9053(R)	Red	200	-
**7.**	2013-9053(B)	Blue	200	-

**Table 2:** Radiocarbon date for a dyed wool fragment from the Hathor Temple (Site 200) *.

ID (ORAU)	ID (IAA)	Age BP	Cal. BCE 1σ		Cal. BCE 2σ		Material
			From	To	From	To	
OxA-30696	2013–9053	2946±25	1208	1121	1227	1052	Textile (wool)

* Dating was done at the University of Oxford’s Radiocarbon Accelerator Unit (ORAU) in the Research Laboratory for Archaeology and the History of Art; calibration by OxCal v. 4.2 and calibration curve IntCal13, copyright: Bronk Ramsey 2013.

### Extraction methods

In order to obtain maximum results without missing cases of double dyeing, which was a very common practice in ancient times [7,29], we used two extraction methods in the course of the study for each of the samples (each sample was divided into two subsamples). The first method used mild extraction that was done using formic acid, a weak acid that preserves the glycosidic linkages and provides good extracting ability. This method is appropriate for the identification of yellow dyes (mainly flavonoid dyes), which are easily decomposed [30,31]. The second method was done using dimethyl sulfoxide (DMSO), a strong organic solvent that enables the complete solubilization of non-flavonoid dyes, such as the red and according to the other studies more effective in the detection the indigoids dyes then other solvent [32,33]:

1. Mixture of formic acid and methanol (5:95, v/v), was heated for 60 min at 50°C with the dyed wool samples. The solution of dissolved color was separated from the wool and transferred to a sterile 1.5 mL microtube. The sample was then dried in a SpeedVac™ (Savant™, Thermo Scientific), causing evaporation of the acid and concentration of the dyes. The residues were then dissolved in 50 μL of methanol/water (1:1, v/v) to produce a clear solution suitable for injection into HPLC.
2. Minute samples from the archaeological textiles were dissolved in 150 μL DMSO, and heated for 10 min at 95°C. The liquid was then separated from the sample via centrifugation and transferred to a sterile Eppendorf 1.5 mL microtube to be processed by HPLC. An amount of 20 μl of the sample was injected.

### HPLC analysis protocol

Extracts of dyed textiles were analyzed by the HPLC-DAD (High-Performance Liquid Chromatography with Diode Array Detector) system (Hitachi LaChrom Elite Chromatography), at the HPLC Unit of the Mina and Everard Goodman Faculty of Life Sciences at Bar-Ilan University. system. HPLC system (running EZ Chrom Elite v. 3.2.1 software) consisted of an L-2130 binary pump, an L-2200 autosampler, an L-2300 column oven (column temperature of 30°C was used for all analyses), and an L-2455 Diode Array Detector, set to obtain chromatogram spectra in the range of 200–700 nm, with extracted chromatograms at 254 nm, 454 nm, and 554 nm. The chromatographic column was a GraceSmart RP18, 5 μm, 250 mm × 4.6 mm ID. Two analytical protocols were used: Protocol A for analysis of red and yellow dyes, and Protocol B for analysis of indigoid dyes (Table 3). The mobile phase for protocol A consisted of a linear gradient of acetonitrile and 100mM ammonium acetate pH4: acetonitrile (9:1 v/v), with the flow rate of 1mL/min and injection volume of 25 μL. The mobile phase for protocol B was made up of A: phosphoric acid 0.5% (w/v), B: methanol and C: H_2_O. The flow rate was held at 1 ml/min and 10 μL injections were made. Gradient elution conditions are tabulated in Table 3. All the samples were analyzed using Protocol A. If indigotin was identified, the samples were submitted for Protocol B analysis, which gives an accurate and quantifiable analysis of the various indigoids and detects Murex-derived dyestuff. The dyes were identified by comparing the results with known standards (S1 Appendix).

**Table 3:** Linear gradient elution of protocols A and B.

Protocol A:		Protocol B:		
**Injection volume:**	**25** μL	**Injection Volume:**	10 μL	
**Solvent A:**	Acetonitrile	Solvent A:	0.5% (w/v) Phosphoric acid	
**Solvent B:**	100mM Ammonium Acetate pH 4: Acetonitrile (9:1)	Solvent B:	Methanol	
		Solvent C:	H_2_O	
**Flow rate:**	1ml/min temp 30°C	1ml/min temp 30°C		
**Time (min)**	% A	Time (min)	% A	% B
0	0	0	10	50
5	0	3	10	80
15	22	20	10	80
30	35	25	10	90
35	35	30	10	90
38	100	33	10	50
40	100	40	10	50
42	0			
50	0			

## Results

**Fig 8:**
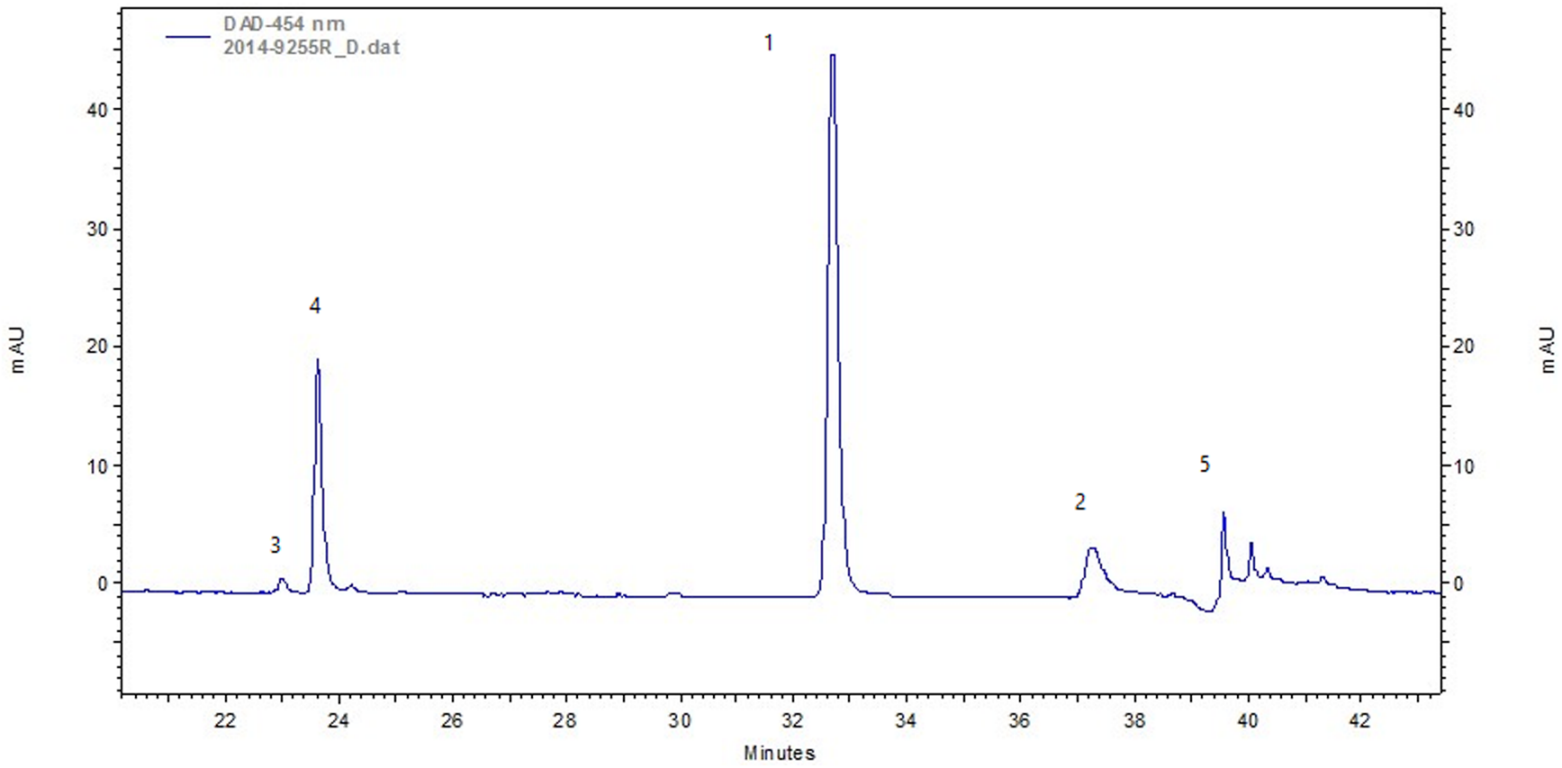
HPLC chromatogram of textile no. 2014-9255R. Extracted using DMSO, wavelength 454 nm. The following components are visible: Peak 1: alizarin (R_t_: 32.32 min); Peak 2: purpurin (R_t_: 24 36.56); Peak 3: pseudopurpurin (R_t_: 23.39); Peak 4: munjistin (R_t_: 24); Peak 5: P2 (R_t_: 39.44).

The analysis results, as obtained using HPLC-DAD instrumentation, are shown in Tables 4 and 5. Compared to the results of known chemical standards and wool dyed with known dyestuffs (S1 Appendix) in their retention time (R_t_) and their absorbance spectra, the compounds in the archaeological dye textile were detected. In all the red samples analyzed according to the two extraction methods, the following components were identified (Table 4, and Fig 8): alizarin at 32 R_t_ (249 nm, 274 nm, and 429 nm λ_max_); purpurin at 37 R_t_ (255 nm, 293 nm, and 481nm λ_max_); pseudopurpurin at 23 R_t_ (258 nm and 493 nm λ_max_), munjistin at 24 R_t_ (247 nm, 287 nm, and 422nm λ_max_), and P2 unknown anthraquinone peak (probably a purpurin derivative), as seen in the chromatogram at 39 R_t_ (240 nm, 258 nm, 501nm λ_max_ [24]; Table A in S1 Appendix). All these anthraquinone components are typical of the madder species [23,34] from the Rubiaceae family, which contains 36 identified components [35] and includes 15 compounds that affect the dyeing process [14]. Determining the particular madder species (*Rubia spp*.) is difficult, as the extraction method and the implemented protocol affect the results of the composition of materials that appear in the chromatography [23,36], as well as influence of the cultivation parameters, harvesting and storage conditions, the age of the plants on the dyes composition and the dyeing process [37]. However, it is possible to see from the chromatogram result a clear trend in the relative amounts of the colorants that make it possible to determine the species of the plant that were used at a high level of probability. In all the results from the red samples, alizarin appears in a compound with a relatively high concentration of the total dye content (more than 54.79%; see fifth column in Table 4, which presents the relative peak areas measured at 454 nm; Fig 8), as is characteristic of *Rubia tinctorum* L. [36]. This may be contrasted with other species known as source of dyestuff in antiquity, such as *R*. *peregrina* L., mainly present in Mediterranean Europe (such as Spain, France, Italy, Greece and England), where the proportion of purpurin is dominant while alizarin appears in relatively minor quantities [23,38–40], or *Galium* species found in temperate regions with similar results [14]. Other species known to be used as a source of dyestuff in antiquity are *R*. *cordifolia* L., also called *R*. *munjistin* L., which was common in South, Southeast and East Asia and had a relatively large amount of the compounds munjistin and pseudopurpurin but minute amounts of alizarin [7,14,36,41], and *R*. *akane* from the Far East, which had a relatively large amount of purpurin but minute amounts of alizarin [7,14,36]. Glycosides as lucidin primeveroside and ruberthric acid were not detected in the red samples from unknown reason, but their presence in the lake depends on the origin and quality of the plants used and on the recipe used for the preparation [42]. Anyway, the presence of glycosides, is of no help in identifying the original plant dyestuff [43].

**Table 4:** The HPLC results on 454 nm of the archaeological red textiles and relative percentages of the peak areas measured, compared to laboratory-dyed sample of *Rubia tinctorum* L.*.

Textile number (IAA)	Retention time (min)	λ_max_ (nm)	Identified	Relative percentages of the main peak areas measured at 454 nm in the chromatograms	Results
**2014–9240**	32.7	249, 279, 429	Alizarin	57.90%	*Rubia tinctorum* L.
	37.31	255, 293, 481	Purpurin	9.13%	
	23.68	258, 494	Pseudopurpurin	10.12%	
	24.23	247, 285, 418	Munjistin	3.02%	
	39.59	240, 258, 501	P2	19.67%	
**2014-9255R**	32.7	250, 279, 429	Alizarin	60.58%	*Rubia tinctorum* L.
	37.3	255, 292, 481	Purpurin	9.47%	
	23.62	258, 493	Pseudopurpurin	19.82%	
	24.21	247, 287, 422	Munjistin	0.61%	
	39.58	258, 499	P2	9.36%	
**2013–9053**	32.45	250, 278, 430	Alizarin	54.97%	*Rubia tinctorum* L.
	36.97	255, 479	Purpurin	11.01%	
	23.51	257, 494	Pseudopurpurin	3.20%	
	24.05	247, 420	Munjistin	0.81%	
	39.5	258, 501	P2	30.72%	
**Dyed wool standard with *R*. *tinctorum* L.**	32.32	249, 274, 428	Alizarin	55.25%	
	36.57	255, 292, 479	Purpurin	11.34%	
	23.39	258, 493	Pseudopurpurin	11.44%	
	24.00	248, 290, 421	Munjistin	13.34%	
	39.44	258, 324, 500	P2	8.31%	

*The results are based on extracts using DMSO and protocol A. The compounds identified by the retention time (R_t_) and their maximum absorption in UV-visible spectrum (λmax). In the fifth column include relative percentages of the main peak areas measured at 454 nm in the chromatograms.

**Table 5:** The HPLC results on 554 nm of the archaeological blue textiles*.

Textile no.	Retention time (min)	λ_max_ (nm)	Identified	Results
**2014–9465**	39.61	240, 285, 608	Indigotin	Indigo plant
	40.38	249, 289, 532	Indirubin	
**2014-9255G**	39.61	240, 285, 608	Indigotin	Indigo plant
	40.37	248, 290, 534	Indirubin	
**823476**	39.60	240, 286, 609	Indigotin	Indigo plant
	40.36	248, 288, 534	Indirubin	
**2013-9053B**	39.46	240, 285, 610	Indigotin	Indigo plant
	40.28	244, 286, 534	Indirubin	

*The results are based on extracts using DMSO and Protocol A, wavelength 554 nm.

The compounds were identified by the retention time (Rt) and their maximum absorption in UV-visible spectrum (λmax) (Table A in S1 Appendix).

**Fig 9:**
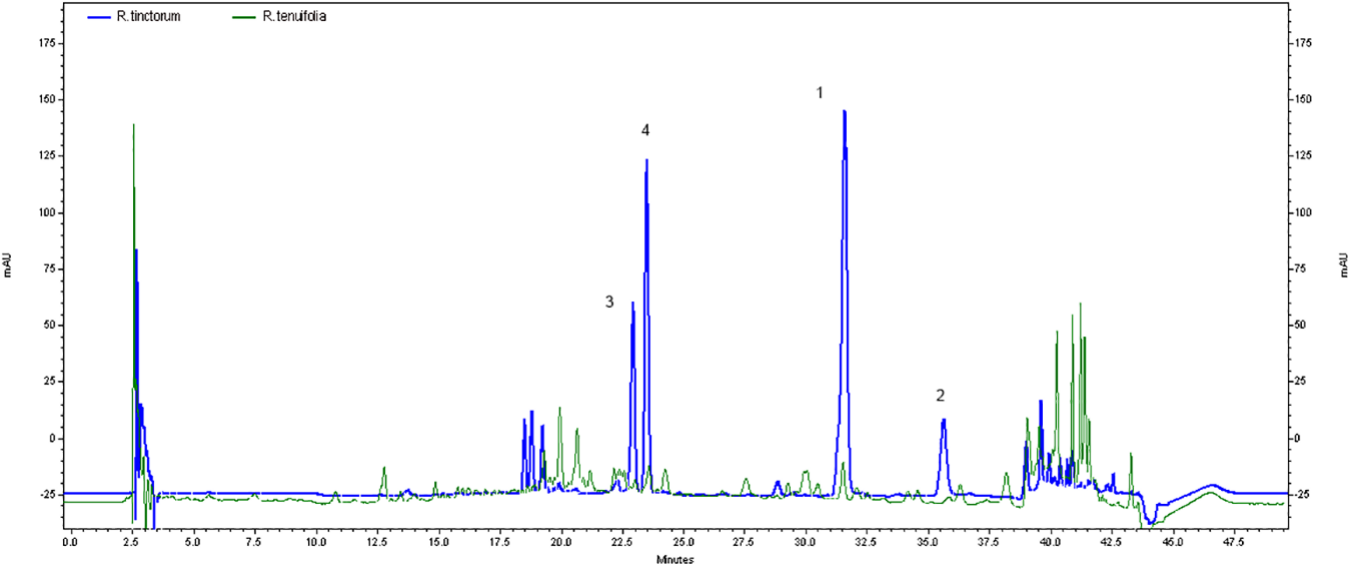
HPLC chromatograms of modern fleece wool dyed with *Rubia tinctorum* L. (blue) vs wool dyed with *Rubia tenuifolia* L. (green). Extracted using DMSO, wavelength 454 nm. The following components are visible: Peak 1: alizarin Peak 2: purpurin; Peak 3: pseudopurpurin; Peak 4: munjistin (R_t_: 24).

**Fig 10:**
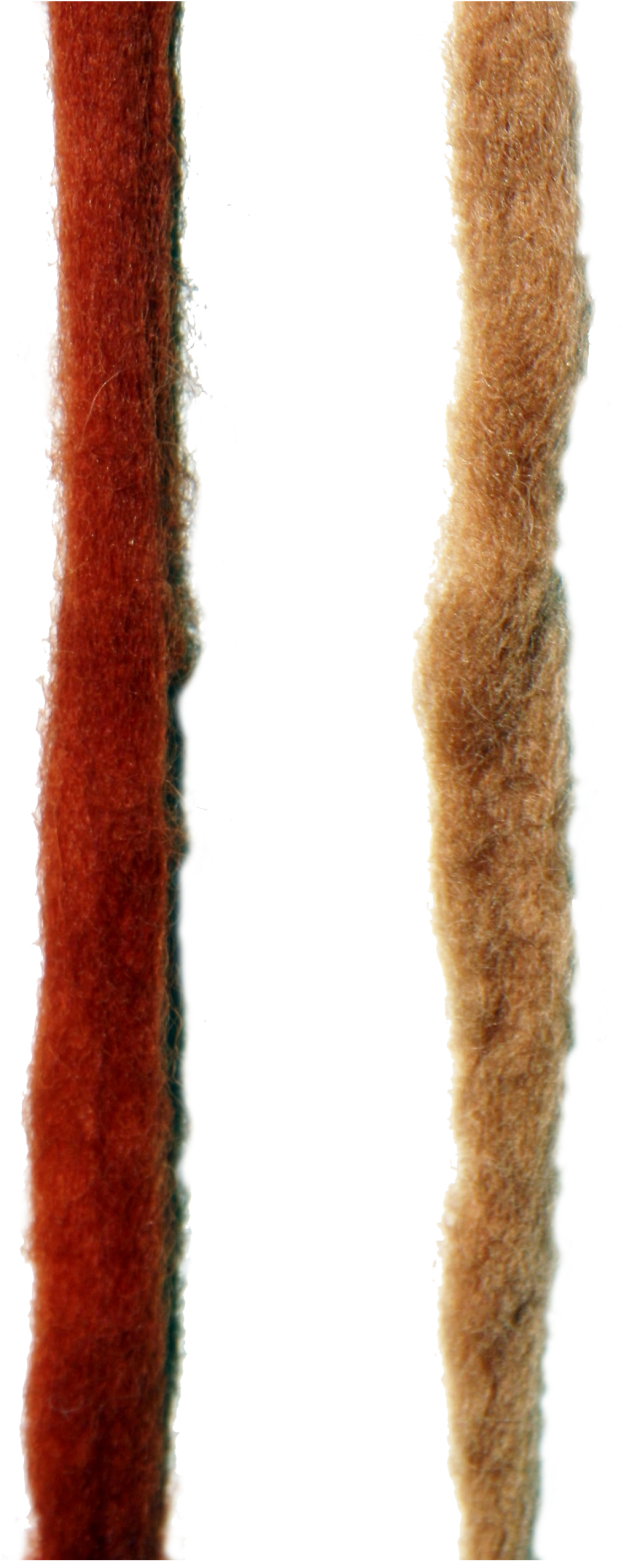
Two modern wool fleeces dyed with madder species. From left to right, wool dyed with *Rubia tinctorum* L., *w*ool dyed with *Rubia tenuifolia* L. Photo by Naama Sukenik.

In order to rule out the possibility of using the local madder species (*R*. *tenuifolia*), which do not have dyeing tradition but grows wild in Israel, as well as in Amon, Moav, and Edom [44], textiles dyed with this plant were analyzed. Reconstruction of dyeing was carried out by our team on wool with *R*. *tenuifolia* L., was analyzed by HPLC. Although the results indicate similar components to those of *Rubia tinctorum* L., containing relatively larger quantities of the alizarin component as compared with the purpurin component [24], the concentration of alizarin was significantly lower than in *Rubia tinctorum* L. (Fig 9). In addition, our reconstruction produced a very light orange-brown color also after long cooking (five hours), that was much lighter and weaker than what appears in the Timna textiles even after thousands of years (Fig 10 and Figs 2 and 3).

**Fig 11:**
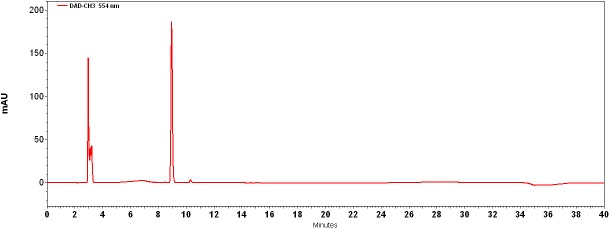
HPLC chromatogram of textile no. 2013–9053 according to protocol B. Extracted using DMSO, wavelength 554 nm. The following components are visible: Peak 1: indigotin (R_t_: 8.87 min); peak 2: indirubin (R_t_: 10.14 min).

The results of analyzing the blue fibers (Table 5, Fig 11; Tables A and B in S1 Appendix), indicate the presence of the indigotin at 39 R_t_ (or 8.87 R_t_ by Protocol B; 240 nm, 285 nm, 609 nm λ_max_) and indirubin at 40 R_t_ (or 10.14 R_t_ by Protocol B; 248 nm, 290 nm, 534 nm λ_max_). These components were found in many indigo plants in the world [14] but in particular from two different species that were known in the ancient world in the Mediterranean area: Woad (*Isatis tinctoria* L.) and indigo (*Indigofera tinctoria* L.) [7,14], additionally, they also were known from species of murex snails that were used to produce the purple dye [45–47]. However, as the results of analysis using Protocol B (which was identical for the elution of the indigoid dyes that may be present in molluskan purple pigments; Table 3; [48]) included no compounds unique to the murex species, such as monobromoindigotin at 12.10 R_t_ or dibromoindigotin at 16.90 R_t_ ([45,46]; Fig 11; Table A in S1 Appendix), therefore, it is possible to determine that our blue textiles were dyed with indigotin from a plant source. As of now, the two possible plants (woad and indigo) cannot be distinguished in modern samples using HPLC, and all the more so in archaeological materials, where the percentage of these components is typically much lower [7,43,49]. The indigotin and the indirubin obtained from either plant from of colorless glycoside of indoxyl precursors. The indigotin formed by the combination of two indoxyl by air oxidation and indirubin produced by the condensation of isatin with indoxl [14]. The most predominant component in the chromatogram is indigotin and the indirubin component appears in relatively small quantities (Fig 11).

Nonetheless, it should be noted that *Isatis tinctoria* L. was already known in the southern Levant since the ancient times, while *Indigofera tinctoria* L., which originated in India, entered into extensive use in the region only after the Islamic conquest [50,51]. However, there is still a possibility that a different species of indigo plant might have been used, such as *Indigofera articulata* or *I*. *coerulea*, which were found in archaeological excavations in Israel, date to the Roman period [52,53], and are appropriate to have produced the blue color.

An extraction method using formic acid was used in an attempt to identify components of yellow dyes in the textile samples. Yellow dyes were commonly used in double dyeing, a technique involving dip-dyeing fibers in two different shades to create varied hues. No spectral pattern of yellow dyes were found based on the most commonly used sources in antiquity [49]. Thus, we may cautiously suggest that the greens seen in textiles such as no. 2014-9055G were most likely originally a blue color and changed the hue as a result of ageing.

## Discussion and conclusions

The art of dyeing textiles is an ancient craft that, according to archaeological evidence, was practiced for thousands of years and probably began to develop during the Neolithic Period, after people started to store seeds and fruits and learned the properties of dyestuffs [54]. The current hypothesis is that initially, different dye materials were used for dyeing the body or for medical purposes, and only later used for dyeing textiles [55]. It seems that in its early stages, the dyeing industry was based only on smearing and mechanically pressing different materials into the textile, and not on “true” dyeing, which results in chemical bonding between the dyes and the fiber [55–57]. These materials included parts of fruits, leaves, soot, blood, and other substances that were not resistant to washing and sun exposure. An example of this can be found in the Chalcolithic (late 5^th^ millennium BCE) textiles from the Cave of the Warrior located near Jericho, where textiles were decorated with black bands made from organic materials like gum, resin, asphalt, or bitumen that were smeared and pressed into the textile [57,58]. In contrast, the textiles found at Timna suggest, for the first time in the Levant, the use of chemical dyeing processes using dyeing plants and an advanced dyeing technique in which a chemical bond between the dyes and the fiber eventually produced a high-quality dyed textile with color that was resistant to laundering and exposure to the sun.

### Madder and woad

**Fig 12:**
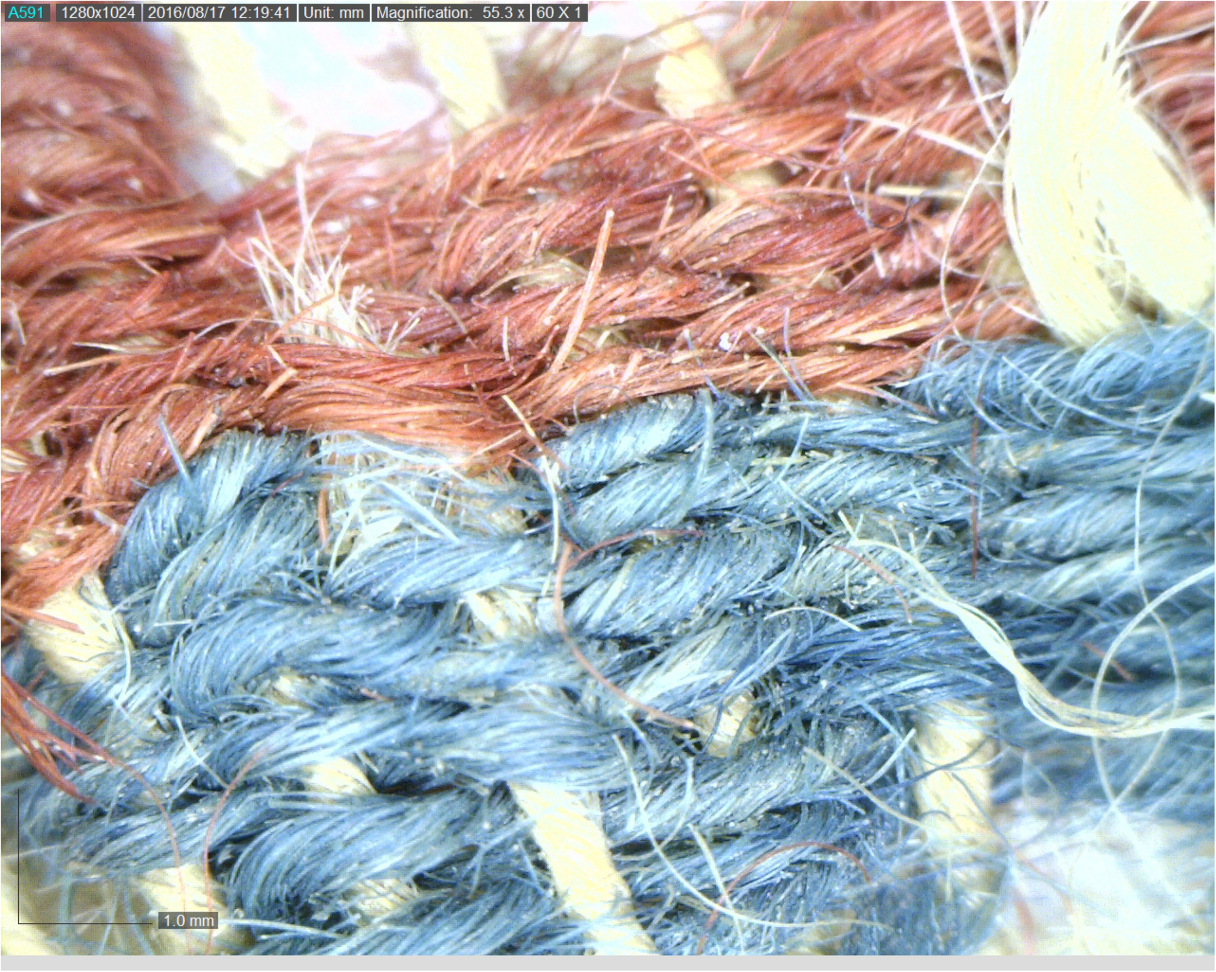
Part of textile IAA no. 2014–9255. According the results, the textile was dyed with madder and plant-based indigotin. Photo by Sukenik using Dino-Lite X 70 magnification.

The results of the current analysis indicated that the textiles at Timna were dyed using two different plants: Madder for red, and plant-based indigotin, probably *Isatis tinctoria* L. (woad), for blue (Fig 12). These plants are among the earliest known in the dyeing craft [50,51,59]. *Rubia tinctorum* L. is a cultured perennial plant, native to the Middle East and Mediterranean region [14]. The dyes concentrated in the long branching roots of the plant had a central role in the ancient dyeing industry of this region, with documented use by the Egyptians, Greeks, and Romans [14]. Although many species of *Rubia* are known [14], only a few of them have been used widely for dyeing in antiquity, including *R*. *peregrine* L., *R*. *cordifolia* L., and *R*. *tinctorum* L. Although theoretically possible that the Timna textiles originated from very distant regions by trade (cf. [60] for trade between the Levant and India at this time), the chemical results indicate that the species of *R*. *tinctorum L*. is the most likely candidate. At the same time, there is still a theoretical possibility that a different species of unknown Rubiaceae have been used.

Madder is one of the mordant dyes [49], which enable a relatively simple process of dyeing by cooking it in water, then adding the wool to the dye solution. For achieving better results, this process was typically accompanied by pretreatment with mordants, which facilitated fixation of the dyes to the fiber. In an attempt to identify mordants used as part of the dyeing process of the Timna textiles we analyzed these textiles by a portable X-ray florescence instrument (Niton XL3t, S2 Appendix). The results show no significant difference to the control group, including undyed textile fragments, experimentally dyed textiles, wool and soil from Timna, thus leading to the conclusion that the amount of aluminum and/or iron resulted from mordants is negligible in relation to background contamination of these abundant elements (especially in soil), and that the identification of the use of mordants and/or their quality cannot be accomplished using this method.

Indigoid plants, identified by the indigotin and indirubin components, are listed among the vat dyes [7], indicating a complex and comprehensive process of reduction and oxidation that took several days in antiquity [14]. In the dyeing process, a solution of leuco-indigo of a greenish-yellow shade was created (soluble reduced form) by producing an alkaline solution by cooking all parts of the plant at a low temperature (30°-40°C) for several days and then adding alkaline materials, *e*.*g*., by potassium carbonate [61], lime, wood ash or by certain plant, such as *Anabasis setifera* or *Mesembryanthemum nodiflorum*, which were used as alkaline material [34,61]. Later is the reduced form of indigotin and forms a yellow water soluble compound. Only after these processes, the wool fleece was dipped into the dye solution. The wool obtained its blue color after being removed from the solution and oxidized in the air [14]. From a chemical viewpoint, suitable conditions (appropriate level of pH, temperature, and oxygen) are created when the leaves of the woad plant are soaked in water during fermentation. The enzymes found in the leaves, a colorless indicant, convert through enzymatic hydrolysis to colorless indoxyl and sugar while the indigotin forms from the combination of two indoxyl molecules by way of oxidation [14]. In any case, dyeing with these plants is more complicated than dyeing with madder, requiring tight control of the temperature and the pH level of the solution, and familiarity with various materials necessary to transform the solution to an alkaline medium. Additionally, the indigotin is reduced by bacterial fermentation–a long process which is often enhanced by adding madder, sugary fruits, treacle, bran, etc. [14,62]. As with madder, the plants were grown primarily for the dyeing industry–in contrast to plants of other dyes and pigments that were also grown for nourishment (*e*.*g*., pomegranates and nuts), for fragrance (*e*.*g*., saffron), and more.

Various historical sources indicate that the plants identified here (madder and plant-based indigotin) were very well-known as a source of dyestuff in later periods. For example, they are mentioned frequently in Greek and Roman natural history literature (for an overview, see [49,63]), which also indicates that the plants were cultivated; similarly, from archeological findings dated to the Roman Period, it emerges that both plants were used intensively in the textile dyeing craft [34,38,40]. Conversely, evidence dated to the Iron Age and earlier periods is scant in both the archaeological and textual regard. In Mesopotamia, madder was known by its Akkadian name *hurratu* in texts from the Larsa region of c.1900 BCE [14]. From texts dated to the Neo-Assyrian Empire (9^th^-7^th^ centuries BCE), it is clear that a dyeing craft based on varied dyestuffs was already known [64]. In a neo-Babylonian tablet dating to the 7^th^ century BCE, two of the oldest recipes appear: one using madder with alum to obtain the red color, and the other describes the double dyeing technique with indigo and madder to imitate the color purple [14]. In the Bible, the name `Pua`(madder in Hebrew) appears as the names of people and family (Num. 26:23) and probably indicates their occupation [65].

The archaeological record is even more limited. In many of the Iron Age sites that yielded textiles, no color fragments were found, including, for example, Kadesh Barnea [66], Tel Deir Alla in Central Jordan Valley [67], and Khirbat al-Mudayna in the southern Transjordan [68]. Owing to the circumstances in which textiles are found, most finds are carbonized and thus the original colors are not preserved. Only few colored textiles were previously recorded: At Site 200 at Timna (the Hathor Temple, also studied here), wool textiles were uncovered that were reported by Rothenberg: “Red and yellow cloth was found… often with beads woven into it” ([28] probably textile no. 2013–9053 of this study). Other red and blue textiles from this site were catalogued in the publication by Sheffer and Tidhar [19] but without dye identification. In addition, other dyed textiles found at Site 30 in Timna (late 12^th^–9^th^ c. BCE) were published only in a preliminary report [69]. In Kuntillat `Ajrud in the eastern Sinai, several dyed textiles were found dating to the Iron Age. Of these, five were linen textiles dyed blue and an additional textile made of blue linen and red wool [15,70,71]. Dye identification indicated that similar to Timna, plant-based indigotin and madder were used to produce the blue and red colors [70]; however, they are dated to the late 8^th^ century BCE ([72,73]. For a slightly earlier date see [74]), two centuries later than the Timna textiles studied here. It should be noted that outside the Levant, plant-based dyed wool textiles were found, for example, in salt mines at Hallstatt (Austria) that date to 1500–1200 BCE (including evidence of, inter alia, indigo plant and plants from the family of Rubiaceae [51,59]). Other early dyed wool textiles were found in Italy dated to the Iron Age [75], and in China’s Xinjiang province at burial sites dated to the early 2nd through the 1st millennium BCE. Also there, HPLC analysis identified madder and plant-based indigotin [43,76]. In Egypt, dyed textiles were found that predate the findings at Timna; these being textiles discovered in the 18^th^ Dynasty in the tomb of Tutankhamon [77,78] and at the Workmen’s Village at Tel el Amarna (around 1350 BCE), where *Rubia tinctorum* L. (madder), and plant-based indigotin were detected [79,80]. Nonetheless, in Egypt, the high quality textiles were predominantly based on linen (including the dyed ones [79]), in contrast to the dyed textiles at Timna, which were made of wool. The blue dyes were suitable for coloring plant fibers, such as linen as well as animal fibers, while the red dyes were less suitable for the former [34]. At any rate, it seems that the dyeing of wool textiles–as reflected in the Timna textiles–developed as part of a larger industry (and related commerce) that specialized in wool and, thus, had a separate evolution than the dyeing of other materials. Based on the available data, the textiles presented here–dated to the late 13^th^-10^th^ centuries BCE–constitute the earliest evidence of plant-based dyed wool from the Levant.

### The origin of the dyed textile

Timna textiles demonstrate improvements not only in dyeing technologies but also in other aspects of textile production. A microscopic analysis of the textiles showed that they were produced with a high level of technical skills exemplified in the expert spinning that created fine, thin threads that were then woven into densely packed weft-faced textiles, and a weaving technique that skillfully executed weft-faced tabby weaves. Additionally, the plying methods (twisting together of spun threads in order to produce thicker and stronger yarn), as well as the low number of textiles with weaving faults found in the collection, are also the mark of an experienced weaver [2]. It seems that the high quality of the color production in the Timna textiles is an integral part of the other technical aspect of the textiles. It should be noted that dyed and finely woven textiles were considered a desired luxury item, conveying the status and wealth of the owner or wearer [81].

*Rubia tinctorum* L. and *Isatis tinctoria* L. (and other plants used for indigotin) are not plants that can grow in Timna. The hyper-arid climate with only 28 mm annual rainfall on average and lack of nearby water sources [82–84] sustains Sudanian vegetation, adapted to these conditions [85], and does not allow such crops of dye plants to grow nor enable even the dyeing process, which requires large quantities of water. This is also true for sheep herding and flax growing which also require more humid conditions; hence, it is evident that the textiles were produced and dyed elsewhere and reached Timna by long-distance transportation and probably trade. Nonetheless, based on historical and geographical considerations, it is reasonable to suggest that the industry existed somewhere in the Levant. The overall high percentage of woolen textiles among the Timna collection may indicate that the source of the fabric is not Egypt, which specialized in growing flax [79], and one must rather seek its source north or northeast of Timna. However, as climatic conditions do not allow the preservation of textiles in most areas of the Levant, it is impossible to reconstruct the source of the Timna textiles based on parallels or any other direct evidence. Thus, such reconstruction has to be based on other means, such as environmental considerations, textual documentations, visual depictions, ethnography and more. Other evidence from the same contexts of the excavated textiles (including botanical and faunal remains) suggests trade connections with the Mediterranean coast and probably with nearby regions such as Judea and Transjordan [3,86], all are possible candidates for the origin of the Timna textiles.

### Insights on the metalworkers society

The results of the dyed textiles research also provide new insights on the social structure and economic status of the Iron Age society at Timna. The high quality textiles and sophisticated dyeing suggest that during the late 11^th^ and 10^th^ centuries BCE (and probably earlier) the people working at Timna were part of a stratified society, which coordinated the tremendous efforts invested in the complex enterprise of copper production in this logistically-challenging region [3,87,88]. The textiles’ close association with contexts directly related to copper smelting [2,3] corroborates results of previous studies that demonstrated the special social status of the metalworkers themselves [89]. The new evidence suggests that the sophisticated task of operating the smelting furnaces was done by skilled metalworkers of high social standing, rather than by a cheap labor force or slaves (as suggested previously [4] and implied by the sites’ common name “Slaves’ Hill”). The high quality dyed textiles were much more desired and expensive than the undyed ones [81], thus their presence suggests the existence of elite at Timna. It is suggested that this elite was part of the ruling class, in which the metalworkers (smelters) took part, or at least were closely associated [3,89]. Metalworkers played a substantial role in ancient societies, holding knowledge of one of the most sophisticated crafts of the ancient world. These included mastery of multiple different parameters, which required skill and precision in order to successfully transform stone into metal [90–92]. Their exalted status, which possibly involved rituals and other cultic activities [93] earned them colored textiles of the highest quality. This is compatible with recent research on the diet of the metalworkers, which points to selected and costly foods prepared especially for the furnace operators; this diet included, among other foods, grapes, pistachios, pomegranates, dates and figs [86,94,95] freshwater and saltwater fish that were brought to Timna from the Mediterranean [89,96,97] and also the best parts of livestock meat like goats and sheep [89,96,98].

To conclude, the study presented here provides the earliest evidence of plant-based textile dyeing from the Levant based on textiles found in a large scale copper smelting site and a nearby temple in the copper ore district of Timna. This evidence, dated to the late 13^th^–10^th^ centuries BCE (with most of the assemblage dated to the late 11^th^–10^th^ c. BCE), not only sheds new light on the development of the textile industry, but also provides insights on social and logistical aspects of copper production in Timna at this formative period, when local kingdoms emerged and replaced the Egyptian hegemony in the region [1].

## Supporting information

S1 Appendix (PDF)

S2 Appendix (PDF)
