# Supplementary material for: Early evidence (late 2nd millennium BCE) of plant-based dyeing of textiles from Timna, Israel
Source: PLoS One. 2017 Jun 28;12(6):e0179014. doi: 10.1371/journal.pone.0179014 (PMC5489155; doi:10.1371/journal.pone.0179014)
Supplement: S1 Appendix — (PDF) [file pone.0179014.s001.pdf]

Appendix A

In order to create a basis for comparison of discovered dyed textiles to known colorants, a database of 37 samples was created and analyzed by HPLC-DAD. The standards include chemically pure compounds from commercial sources and wool fleece that was dyed in the laboratory with known dyestuffs and analyzed by the authors as part of a comprehensive study of dyed archaeological textiles undertaken since 2013 [34,46,99]. According to the historical literature and documentary evidence, those natural dyestuffs were the main dyes used in antiquity (for more details [34,49]). The standards were analyzed using the same methods that most probably had been used for the archaeological samples (described in detail above, see Materials & Methods). All the wool samples were processed using the corresponding extraction procedure (formic acid or DMSO).

HPLC-DAD analysis of the red and yellow dye samples was performed according to Protocol A and the blue-purple dyes were analyzed using Protocol B (the results are marked with asterisks in Table A). The chemical standards were dissolved with methanol and water (1:1, v/v) (excluding indigotin and indirubin, which were dissolved with DMSO), and analyzed according to Protocol A. In each dyed-wool sample, the characteristic chromatogram was obtained, and the constituent colorants were identified according to their retention time (R_t) and absorbance spectra, including the wavelengths of absorbance peaks in the UV-visible spectrum (λmax). Experimental results of the dyed wool analyses are summarized in Table A. Confirmation of the identification of the compounds was obtained by comparison with known pure standards, as listed in Table B. All the chemical standards were purchased from Sigma-Aldrich: alizarin (no. 122777), purpurin (no. 229148), apigenin (no. A3145), juglone (no. H47003), indirubin (no. 10404), indigotin (no. 229296), lawsone (no. H46805), brazlin (no. 633649), kaempferol (no. 60010), carminic acid (no. 11298), ellagic acid (14668), crocin (no. 17304), isatin (no. 58240), and laccaic acid A and B (no. 50506). In a few cases when the necessary standard was not available, dyed wool fleece was analyzed, and constituent colorants were identified and compared with literature data from similar studies.

The natural dyestuffs (of plant and animal origin) used were obtained from the collection of Prof. Zohar Amar, Bar Ilan University, and included organic material collected from Israel and from all over the world. Reconstruction of dyeing was carried out on wool, the main raw material dyed in textiles at Timna [2] and other sites in ancient times [10,38,99] and according to protocols for dyeing with natural colorants (the dyeing procedure is described in detail in [34]). In most cases, dyeing procedure included the same steps: the dyestuff materials were crushed and heated to 70°C in tap water for at least half an hour. After obtaining a color solution, wool samples were cooked for a least one hour. The mordant dyes (*Rubia tinctorium* L., *Caesalpinia sappan* L., *Kermes echinatus, Kermes vermilio, Dactylopius coccus, Porphyrophora hamelii*, *Laccifer Lacca*, *Carthamus tinctorius* L., *Reseda luteola* L., *Alkanna tinctoria* L., *Chrozophora tinctoria* L., and *Roccella tinctoria* L.) need wool pretreated with alum. The indigoid dyes (including *Isatis tinctoria* L, *Indigofera tinctoria* L., and Murex species) were prepared in the laboratory as follows: Three grams of sodium hydroxide was added to 0.2-3 grams of crushed dyestuff in 200 ml tap water and the solution was stirred. Twelve grams of sodium hydrosulfite ($Na_2S_2O_4$) was then mixed in and stirred slowly to avoid oxygenation, followed by 4.5 grams of household-grade

citric acid. The wool sample was then placed in a beaker with the dyeing solution and incubated for at least 15 minutes before it was removed and exposed to air. To achieve red color from *Carthamus tinctorius* L. (safflower), petals were first washed with water until the yellow dyes was completely removed. Then, sodium carbonate was added to the solution with water until pH 10.5 was reached. After one hour, citric acid (10%) was added to obtain pH of 3.5-4 and the threads (made of silk or linen) were then placed in the solution.

Table A lists retention time (Rt) and light absorbance maxima in UV-visible spectrum (λmax) of known dyed wool (categorized according to their colors) and Table B lists the same properties of known chemical standards. This data could serve as a reference for future studies.

Table A: HPLC analysis of known dyed wool using HPLC-DAD

	Dye source	Detected component	Rt (min) according to the extraction methods		λmax (nm)	Reference and comments
			Formic acid	DMSO using protocol A / * Refers to Rt using Protocol B		
Red dyes	*Rubia tinctorium* L.<br>*Rubia tenuifolia* L.<br>*Rubia cordifolia* L.	Alizarin	33.0	32.32	249, 274, 428	See Table B [42]
		Purpurin	39.2	36.57	255, 292, 479	See Table B [42]
		Pseudopurpurin		23.39	258, 493	[42]
		Munjistin	24.36	24.00	248, 290, 421	[42,100]
		P2		39.44	258, 324, 500	Without parallel
	Caesalpinia sappan L.	Brazilin	18.54	18.10	236, 271, 445	See Table B
	Juglans regia L.	Juglone	26.97	26.28	264, 423	See Table B
	Lawsonia inermis L.	Luteolin	24.03	23.56	254, 348	See Table B [101]
		Apigenin	28.0	27.47	242, 266, 337	
		Lawsone	28.65	27.89	272, 333, 432	
	Kermes echinatus/ Kermes vermilio	Kermesic acid	18.94	18.94	236, 278, 485	[102]
		Flavokermesic acid	19.0	19.10	231, 288, 433	

	Dactylopius coccus Porphyrophora hamelii	Carminic acid	11.47	11.93	234, 278, 492	[102,103]
		Kermesic acid	15.97	16.2	240, 280, 487	
		Flavokermesic acid	18.72	18.9	246, 288, 434	
		C1	7.71	11.5	280, 288, 332, 513, 554	
	Laccifer Lacca	Laccaic acid A Laccaic acid B	17.53<br>17.54	17.04<br>16.81	238, 288, 490<br>240, 288, 488	See Table B
	Carthamus tinctorius L.	Red Carthamin	24.81	24.68	247, 372, 520	[104]
Yellow dyes		Safflower yellow	18.74	18.30	243, 409	[100]; Similar peaks were seen in the chromatogram.
		S1	32.47	31.67	243, 281, 367	
	Reseda luteola L.	Luteolin	17.64	17.36	255, 348	[100,101,105]
		Apigenin	19.16	18.88	240, 266, 337	See Table B
	Crocus sativus L.	Crocin	28.69	28.23	258, 435, 458	See Table B; Similar peaks were seen in the chromatogram.
	Punica granatum L.	Ellagic acid	16.72	16.82	254, 366	See Table B
	Galls (Quercus ithaburensis, Q. infectoria, Q. lusitanica	Ellagic acid	17.21	17.10	254, 366	
Blue – purple dyes	*Alkanna tinctoria* L.	Alkannin	42.19	42.08	273, 490, 516	[100,106]; Similar spectrum appears after 43 min.
	Chrozophora tinctoria L.	C	16.40	16.22	240, 329, 569	[107]
		T2	18.40	18.21	240, 327, 576	
	Roccella tinctoria L.	Orcein	32.10	32.86	242, 540, 559	[108]
	Isatis tinctoria L.	Indigotin	39.8	39.32/<br>8.87*	241, 286, 611	See Table B
		Indirubin	40.25	40.24/<br>10.14*	289, 362, 540	See Table B
		Isatin	13.43	13.46/<br>4.24*	241, 300,	[45]
	Indigofera tinctoria L.	Indigotin	39.8	39.32/<br>8.87*	241, 286, 611	See Table B [45]

		Indirubin	40.25	40.24<br>10.14*	289, 362, 542	See Table B [45]
		Isatin	13.43	13.30/<br>4.24*	241, 300, 421	See Table B [45]
	Hexaplex trunculus Bolinus brandaris Stramonita haemastoma	Isatin	13.30	13.46/<br>4.30*	241, 301, 417	[45]
		Indigotin	38.8	39.32/<br>9.12*	241, 286, 613	[45]
		Indirubin	40.23	40.26/<br>10.51*	289, 363, 542	[45]
		Monobromoindigotin	---	41.52/<br>12.10*	242, 289, 348, 608	[45]
		Monobromoindirubin	---	41.79/<br>14.81*	243, 298, 366, 536	[45]
		Dibromoindigotin	---	42.59/<br>16. 90*	292, 303, 351, 601	[45]
		Dibromoindirubin	---	42.95/<br>24.94*	254, 301, 368, 544	[45]

Table B: Analyses of chemical standards using HPLC-DAD. Identification by retention time (Rt) and their maximum absorption in UV-visible spectrum (λmax)

Standards	Retention time (min)	max (nm)
Alizarin	32.35	250, 279, 429
Apigenin	27.21	239, 266, 388
Brazilin	17.87	243, 445
Carminic acid	11.81	236, 277, 492
Crocin	27.63	435, 459
Ellagic acid	16.76	253, 363
Indigotin	39.36	242, 286, 611
Indirubin	40.34	246, 290, 541
Isatin	13.53	243, 302, 421
Juglone	26.16	253, 424
Laccaic acid A<br>Laccaic acid B	16.68<br>16.46	289, 491
Lawsone	27.85	252, 270, 334, 423
Purpurin	36.31	255, 293, 481
