# Supplementary material for: Early evidence (late 2nd millennium BCE) of plant-based dyeing of textiles from Timna, Israel
Source: PLoS One. 2017 Jun 28;12(6):e0179014. doi: 10.1371/journal.pone.0179014 (PMC5489155; doi:10.1371/journal.pone.0179014)
Supplement: S2 Appendix — (PDF) [file pone.0179014.s002.pdf]

Appendix B

In an attempt to identify the mordents that were used in the Timna textiles we applied X-ray fluorescence (XRF) analysis on ancient and modern textile fragments, dyed and undyed, as well as on soil samples from Timna. We used a Niton XL3t portable XRF instrument, measuring each samples for 90 seconds with calibration setting sensitive to the elements of interest (Fe, Al; "Mining" operation mode). The results are shown in Table A.

Six different ancient textiles from Timna were analyzed (readings nos. 1-13) at least in two different spots (marked: (1) / (2) in Table A); textiles no. 2014-9255/3 (reading no. 11) and no. 2015-1900 (readings nos. 12-13) were undyed and textile no. 2014-2955/2 (reading no. 10) was dyed blue (vat dye, without mordants). The red color in textiles nos. 2013-9053/1, 2014-9240 and 2014-9255/1 were detected in the current research as *Rubia tinctorum* L. that belong to the mordant dye and hence mordants are expected.

For control, we analyzed two samples of soil from Timna (readings nos. 14-15); clean wool (readings nos. 16-17) and various modern dyed wool: three different fleeces that were pretreated with alum ($KAl(SO_4)_2 \cdot 12H_2O$) and were dyed with madder (*Rubia tinctorum* L. and *R. tenuifolia* L.; see samples nos. 18-23); fleece that was dyed with *Isatis tinctoria* L. without mordant (see S1 Appendix A; readings nos. 24-25); fleece that was dyed with hematite (iron oxide) and alum (readings nos. 26-27); fleece that was dyed with madder alum and iron (readings nos. 26-27) and fleece that was dyed with madder alum and copper.

The results (Table A) indicate the presence of iron, aluminum, potassium, calcium, silicon, sulfide and copper. Similar concentrations of these elements, and in particular of iron and aluminum (the main components of potential mordants), were found in the dyed and undyed textiles (modern and ancient) and the soil samples from Timna, suggesting that the amount of aluminum and/or iron resulted from mordants is negligible in relation to background contamination of these abundant elements (especially in soil). Thus, it seems that XRF analysis cannot be used to decisively determine the use of mordant in ancient dyed textiles, and previous results based on such analysis should be taken cautiously [38].

The relatively high concentrations of iron in textile no. 2014-9240 (readings nos. 3-4) might indicate the use of hematite as pigment [109,110]. However, in the modern textile that was dyed with madder and hematite (readings nos. 26-27), much less iron was detected, further stressing the difficulty of using direct chemical analysis for assessing the use of mordants.

Table A: Results of XRF

Reading No.	Sample	Source	Description	Main elements (wt.%)							
				Fe	Al	K	Ca	Si	Cl	Cu	S
1	textile-2013-9053 (1)	Timna textile	red textile (detected with *R. tinctorum* L.)	1.80	1.07	1.23	10.03	5.38	1.04	0.07	8.28
2	textile-2013-9053 (2)	Timna textile	red textile (detected with *R. tinctorum* L.)	1.61	0.97	1.30	11.91	4.57	1.10	0.06	8.73
3	textile-2014-9240 (1)	Timna textile	red textile (detected with *R. tinctorum* L.)	6.64	4.90	3.67	18.64	12.70	1.44	2.10	8.56
4	textile-2014-9240 (2)	Timna textile	red textile (detected with *R. tinctorum* L.)	5.73	3.93	4.86	17.95	9.74	1.88	2.10	11.90
5	textile-2014-9257 (1)	Timna textile	red textile	2.59	5.11	7.71	12.98	8.49	20.36	1.11	12.71
6	textile-2014-9257 (2)	Timna textile	red textile	2.78	3.51	8.58	14.41	6.57	15.93	1.00	18.57
7	textile-2014-9465 (1)	Timna textile	red textile	3.53	5.48	7.52	6.64	11.38	11.18	0.83	14.27
8	textile-2014-9465 (2)	Timna textile	red textile	2.96	4.40	7.01	7.74	9.96	9.59	0.81	12.69
9	textile-2014-9255 (1)	Timna textile	red textile (detected with *R. tinctorum* L.)	1.97	1.41	3.99	10.80	4.16	7.58	0.59	20.06
10	textile-2014-9255 (2)	Timna textile	blue textile (detected with indigotin)	2.41	1.94	3.36	11.65	4.18	8.10	0.54	22.92
11	textile-2014-9255 (3)	Timna textile	undyed textile	1.81	2.37	5.07	11.55	3.88	10.50	0.45	29.43
12	textile-2015-1900 (1)	Timna textile	undyed textile	3.92	2.94	7.75	13.69	8.51	2.52	1.41	9.85
13	textile-2015-1900 (2)	Timna textile	undyed textile	3.78	2.21	8.13	13.85	7.33	3.25	1.43	10.44
14	soil from Timna	modern	sample no. 736 (Site 34, surface)	0.93	4.23	0.38	5.04	17.97	0.00	0.06	0.39
15	soil from Timna	modern	sample no. 738 (Site 34, surface)	1.71	3.51	0.61	8.53	16.18	0.08	0.05	0.08
16	wool (1)	modern	clean wool	1.25	0.38	0.02	0.46	1.42	0.32	0.08	18.02
17	wool (2)	modern	clean wool	2.46	0.42	0.07	0.79	2.22	0.60	0.08	26.24
18	wool-madder-alum- A (1)	modern	dyed wool with *Rubia tinctorum* L. and alum	4.49	1.00	0.19	1.40	4.24	1.11	0.23	21.31

19	wool-madder-alum- A (2)	modern	dyed wool with *Rubia tinctorum* L. and alum	3.66	0.90	0.17	1.47	3.57	1.19	0.19	20.55
20	wool-madder-alum- B (1)	modern	dyed wool with *Rubia tinctorum* L. and alum	1.29	0.50	0.02	0.97	1.69	0.39	0.05	17.74
21	wool-madder-alum- B (2)	modern	dyed wool with *Rubia tinctorum* L. and alum	1.27	0.26	0.13	0.86	1.95	0.41	0.06	14.40
22	wool-madder-alum- C (1)	modern	dyed wool with *Rubia tinctorum* L. and alum	0.31	0.27	0.18	0.66	0.70	4.01	0.02	10.78
23	wool-madder-alum- C (2)	modern	dyed wool with *Rubia tinctorum* L. and alum	0.30	0.25	0.07	0.74	0.91	0.38	0.03	11.57
24	wool-isatis (1)	modern	dyed wool with *Isatis tinctoria L.*	0.35	0.17	0.02	1.48	1.05	0.54	0.03	15.07
25	wool-isatis (2)	modern	dyed wool with *Isatis tinctoria L.*	0.42	0.26	0.02	1.43	1.16	0.47	0.04	13.56
26	wool-hematite-alum (1)	modern	dyed wool with hematite and alum	0.90	0.21	0.01	1.49	0.43	0.33	0.00	16.31
27	wool-hematite-alum (2)	modern	dyed wool with hematite and alum	0.89	0.25	0.02	1.65	1.05	0.47	0.01	18.65
28	wool-madder-alum-iron (1)	modern	dyed wool with *Rubia tinctorum* L. and iron	1.06	1.21	0.09	1.84	2.85	1.37	0.10	49.37
29	wool-madder-alum-iron (2)	modern	dyed wool with *Rubia tinctorum* L. and iron	0.88	0.52	0.05	1.38	2.64	1.02	0.11	36.35
30	wool-madder-alum-copper (1)	modern	dyed wool with *Rubia tinctorum* L. and copper	0.29	0.42	0.04	0.58	1.09	0.96	0.01	34.96
31	wool-madder-alum-copper (2)	modern	dyed wool with *Rubia tinctorum* L. and copper	0.27	0.53	0.07	0.71	0.96	1.13	0.01	36.65
